# Remdesivir for COVID‐19 pneumonia in patients with severe chronic kidney disease: A Case series and review of the literature

**DOI:** 10.1002/ccr3.5467

**Published:** 2022-02-24

**Authors:** Ahmad Al Bishawi, Hamad Abdel Hadi, Eman Elmekaty, Musaed Al Samawi, Arun Nair, Mohammed Abou Kamar, Muna Al Maslamani, Alaaeldin Abdelmajid

**Affiliations:** ^1^ 36977 Division of infectious Diseases Department of Internal Medicine Hamad Medical Corporation Doha Qatar; ^2^ 36977 Division of infectious Diseases Department of Clinical Pharmacy Hamad Medical Corporation Doha Qatar

**Keywords:** chronic kidney diseases (CKD), COVID‐19, remdesivir, SARS‐CoV‐2

## Abstract

Remdesivir was the first antiviral agent to receive FDA authorization for severe COVID‐19 management, which restricts its use with severe renal impairment due to concerns that active metabolites might accumulate, causing renal toxicities. With limited treatment options, available evidence on such patient groups is important to assess for future safety.

## Abstract 2

Our report suggests that Remdesivir is well tolerated and potentially safe among hospitalized patients with severe COVID‐19 infection with the background of advanced kidney diseases where benefits outweigh potential risks.

## 1 INTRODUCTION

In late 2019, SARS‐CoV‐2 infection leading to COVID‐19 disease emerged from Wuhan province in China to cause an unprecedented pandemic with global consequences. From foremost reports, older patients with chronic conditions including chronic kidney diseases (CKD) were at increased risks for COVID‐19 progression and complications. Despite the unparallel scientific race to develop novel therapeutic agents, there is paucity of effective treatment options. Remdesivir, a competitive RNA nucleoside polymerase inhibitor that terminates viral replications, was the first drug to receive approval from the FDA in the USA under the auspices of emergency use authorization for the management of hospitalized patients with moderate to severe COVID‐19 disease. Since its approval, clinical trials excluded patients with advanced CKD or patients on dialysis, as the approval did not cover such cohort of patients. Nevertheless, such cohort of patients are vulnerable for severe COVID‐19 disease, and in the absence of other effective management at the start of the pandemic, no other alternative options were available. Clinical teams were forced to cautiously evaluate its use in such vulnerable groups and then share the experience for the benefit of the scientific community.

In this case series of hospitalized patients with moderate to severe COVID‐19 disease and advanced CKD were treated with Remdesivir, we aim to highlight the safety and tolerability profiles of this therapeutic agent in patients with advanced CKD.

## 2 CASE PRESENTATIONS, DEMOGRAPHIC, AND CLINICAL CHARACTERIZATION SUMMARY

### 2.1 Case 1

A 63‐year‐old man with a background history of renal transplant in 2014 from living related donor maintained on sirolimus and cyclosporin with chronic graft dysfunction (CKD stage 4) and baseline creatinine of 220 μmol/L, presented in June 2020 with fever, cough, and dyspnea eventually diagnosed as severe COVID‐19 pneumonia requiring oxygen therapy (Table 1). Because of vulnerability and limited alternative treatment options, the patient was evaluated for combined Remdesivir and dexamethasone treatment and went to receive outlined management successfully. During hospital course, the highest recorded creatinine was 294 μmol/L, which responded to fluid support and returned to baseline value. He was later discharged home safely within 2 weeks from hospital admission.

**TABLE 1:** Demographic and clinical characteristics of the patients

Patient	Age	Gender	Co‐morbidities	Presenting symptoms	Radiological findings	Oxygen support
Case 1	63 years	Male	Living donor renal transplant	Fever, dry cough, dyspnea	Upper mid and lower ground glass opacities	3 liters/min
Case 2	61 years	Male	Coronary artery Disease, Hypertension, End stage renal disease secondary to Polycystic Kidney and Diabetes mellitus, Cadaveric Renal transplant	Dry cough, headache, body ache, fever	Disseminated bilateral ground glass appearance and inflammatory lesions	5 liters/min, then 7 liters/min
Case 3	32 years	Female	Chronic Kidney Disease to Diabetic Nephropathy	Cough, fever, body ache	Right lower and mid ground glass opacities	4–5 liters/min
Case 4	92 years	Female	Diabetes mellitus and essential hypertension, CKD	Cough, Fever, abdominal pain, fatigue	Upper mid and lower ground glass opacities	1 liter/min, then 12–15 L/min, reaching up to 60 L/min
Case 5	88 years	Male	Diabetes mellitus, hypertension, prostate cancer, coronary artery disease, CKD	Cough and fever	Showed bilateral lower infiltrates and faint ground glass opacity in the peripheries	1–2 liters/min

### 2.2 Case 2

A 61‐year‐old man with a background history of adult polycystic kidney disease and a history of renal transplant from cadaveric donor in 2015 maintained on azathioprine, prednisolone, and tacrolimus with chronic graft dysfunction (CKD stage 4) and baseline creatinine of 238 μmol/L. In February 2020, he developed mild COVID‐19 infection and recovered without any residual complications. Six months later in August 2020, the patient presented with fever and dyspnea eventually diagnosed as COVID‐19 pneumonia and started on oral Favipiravir as per local protocol. One week into hospital admission, the patient's condition worsened with rising serum creatinine to 261 μmol/L and increased oxygen requirements. To avoid further deterioration, the patient was evaluated and received successfully combined Remdesivir and dexamethasone therapy. He had a gradual and steady clinical improvement with creatinine falling to nadir 205 μmol/L, then was discharged from hospital on Day 16.

### 2.3 Case 3

A 32‐year‐old woman with advanced CKD secondary to diabetic nephropathy (CKD stage 4) with baseline serum creatinine of 332 mmol/L was admitted with respiratory symptoms of COVID‐19 pneumonia to receive oxygen due to hypoxemia. Upon initial assessment, she had worsening of renal functions with oliguria and rising serum creatinine up to 891 μmol/L, requiring two sessions of hemodialysis. To avert further complications, she was started on Remdesivir combined with dexamethasone. With outlined interventions, her overall clinical condition and renal functions improved gradually requiring no further dialysis sessions and returning to baseline value at Day 10, and was discharged safely to home.

### 2.4 Case 4

A 92‐year‐old woman with a background history of diabetes and hypertension with stable kidney parameters with baseline creatinine of 81 μmol/L (CKD stage 3B) upon recent evaluation. She was admitted with mild COVID‐19 pneumonia but deteriorated rapidly, necessitating intensive care unit (ICU) admission culminating into acute respiratory distress syndrome (ARDS) in need of high oxygen requirements. Her course was further complicated by acute kidney injury (AKI) with serum creatinine of 134 μmol/L. Because of her significant deterioration, she was evaluated per the local protocol to receive combined Remdesivir and dexamethasone. Her hospital ICU course was initially complicated, and her clinical state was fluctuating. She received one dose of Tocilizumab for cytokine storm feature, eventually, her renal function gradually improved and returned to baseline values. She was subsequently safely discharged on Day 12 from admission.

### 2.5 Case 5

An 88‐year‐old man with a history of CKD (CKD stage 4) secondary to diabetic nephropathy, with baseline serum creatinine of 123 μmol/L, presented with confirmed mild COVID‐19 disease. Since the patient was not hypoxic, an alternative regimen including favipiravir treatment was instituted initially. However, he rapidly deteriorated requiring supplementary oxygen therapy with early signs of cytokine storm. Because of his age, comorbidities, and disease severity, the patient was evaluated to receive local protocol of Remdesivir and dexamethasone together with tocilizumab. With the timely interventions, the patient's symptoms improved remarkably, and within 6 days he required no supplemental oxygen therapy. Upon discharge, his kidney function almost went back to normal with serum creatinine of 126.

## 3 DISCUSSION

In March 2020, the WHO declared SARS‐CoV‐2 and COVID‐19 disease as a global pandemic which led to catastrophic clinical, social, and economic consequences.^1^, ^2^ The spectrum of the disease is intriguingly variable ranging from asymptomatic infection, mild to moderate, severe, culminating to stormy life‐threatening multiorgan failure leading to significant morbidity and mortality.^3^ Since the unique disease caught the unprepared healthcare settings across the globe by a surprise, there were no available effective therapeutic agents, which resulted in a race for the international medical and scientific communities to discover effective therapy.^4^

To date, many observational studies suggested the use of certain drugs in the treatment regimen for COVID‐19 patients, including but not limited to (Steroid, ACE inhibitors, Statins, immunomodulatory agents, Lopinovir/Ritonavir, Favipiravir, and convalescent Plasma).^5^ Nevertheless, there is no conclusively proven curative therapy for COVID‐19 as of now.

Among many of the first studied repurposed antiviral agents were Remdesivir. The pro‐drug, which previously demonstrated comparable efficacy against similar RNA viruses, is a nucleoside analog that inhibits viral RNA‐dependent polymerase, inhibiting viral replication through chain termination.^6^

Based on the availability of clinical evidence from randomized clinical trials to treat hospitalized COVID‐19 patients with severe disease, the Food and Drug Agency in the US granted emergency authorization for the drug in May 2020, for use for moderate to severe COVID‐19‐related pneumonia. Although Remdesivir did not demonstrate mortality benefits, its efficacy in decreasing recovery time in moderate and severe COVID‐19 infection has been highlighted during large scale randomized clinical trials.^7^

Despite highlighting the evidence‐based background, it must be emphasized that the COVID‐19 pandemic witnessed an unprecedented conflicting scientific evidence where the interpretation of available evidence and efficacy has been greatly debated. While some reviews and meta‐analysis supported Remdesivir therapy for hospitalized patients with COVID‐19 disease, others were skeptical.^8^, ^9^ Nevertheless, the drug continued to bear FDA emergency approval but based on fears of deleterious adverse events in patients with severe CKD, in their licensing authorization, the FDA recommended limiting its use for patients with eGFR less than 30 mL/min unless potential benefits outweigh risks.^10^

The background pathophysiology stems from the theoretical concern that the drug active byproduct metabolite—sulfobutylether beta‐cyclodextrin—might accumulate and cause potential nephro‐toxicities since patients with advanced CKD were excluded from evaluation trials.^11^ Contrary to the precautionary advice, for patients with eGFR <30 some reported studies recommend dose adjustment rather than precautionary limits.^12^ On the other hand, animal studies demonstrated potential, beneficial, renal effects in reversing obstructive renal fibrosis.^13^ Of note, such cohort of patients with chronic kidney disease at significant risks of acquisition and complications of COVID‐19 disease as highlighted from multiple observational studies.^14^ Such circumstances require developing management strategies for such population including careful evaluation, trials of therapy then sharing the experience.

This case series presents five different cases of patients with advanced renal disease who were hospitalized following the diagnosis of COVID‐19 disease then evaluated to receive Remdesivir which was eventually safely administered without significant adverse outcomes. All patients had PCR confirmed COVID‐19 disease with pulmonary involvement, which was radiologically established and required oxygen supplementations. The patients’ spectrum and characteristics were variable in terms of age, comorbidities, underlying cause of CKD, baseline kidney functions, and hospital course. Nevertheless, following careful evaluation of the need for therapeutic interventions, all patients received Remdesivir safely without significant deterioration in their renal functions and eventually fully recovered from their COVID‐19 disease with no harmful outcomes (Table 2). To avoid confounding measures, it must be highlighted that all patients also received dexamethasone, one of the few non‐controversial beneficial COVID‐19 adjuvant management agents that proven to affect both morbidity and mortality in hospitalized patients.^15^ It is quite possible that both safety and tolerability of Remdesivir was partially affected by the co‐administration of the immune modulating therapy that has protective functions.

**TABLE 2:** Kidney function and treatment outcome

Patient	Baseline serum creatinine (μmol/L)	Admission highest serum creatinine (μmol/L)	Admission highest blood urea (mmol/L)	Lowest admission eGFR (ml/min)	Discharge serum creatinine (μmol/L)	Hospitalization days	Treatment outcome
Case 1	220	294	17.2	15	226	14	Improved, discharged
Case 2	238	261	13.6	22	205	16	Improved, discharged
Case 3	332	891	33.7	10	340	10	Required 2 sessions of hemodialysis, then improved, discharged
Case 4	81	134	15.7	20	85	12	Required ICU admission, then improved and discharged home
Case 5	123	123	11.3	26	126	7	Improved, discharged

In the absence of supporting clinical data and guidelines for the appropriate management of patient with COVID‐19 disease and advanced CKD including Remdesivir, it might defer clinicians from administering the limited available therapeutic options particularly if there are licensing precautions. For example, only limited studies evaluated Remdesivir in the context of acute or chronic renal disease and highlighted management interventions. A retrospective evaluation of 109 elderly patients almost half of them had CKD stage 3, demonstrated overall safety and tolerability.^16^

Similarly, a study of 46 patients with acute kidney injury or advanced CKD including patients on dialysis admitted to renal ICU, received Remdesivir with no monitored deterioration and conclusive evidence of safety.^17^ Conversely, 7 patients with kidney transplant received the drug without harmful outcomes.^18^ These studies support our experience that Remdesivir can be safely administered in patients with advanced CKD including transplant recipients.

## 4 CONCLUSION

For patients with advanced kidney disease, precautions against the use Remdesivir in this population might hinder limited available treatment options. There is an accumulating supporting evidence of the safety and tolerability of the drug which certainly need wider evaluation.

## CONFLICT OF INTEREST

No conflict of interest declared by authors.

## AUTHOR CONTRIBUTIONS

Ahmad Al Bishawi involved in conceptualization, writing – original draft, data curation. Hamad Abdelhadi involved in conceptualization, data analysis, writing – review & editing, supervision. Alaaeldin Abdelmajid involved in data collection, data analysis, manuscript writing, supervision. Eman Elmekaty involved in data collection, data analysis, manuscript writing. Musaed Al Samawi involved in data collection. Arun Nair involved in data collection. Mohammed Abou Kamar involved in data collection. Muna Al Maslamani involved in conceptualization, writing – review & editing, supervision.

## ETHICAL APPROVAL

Consent form was obtained from the patients towards academic publication. The case report received approval from HMC medical research center (MRC) for publication under MRC‐0421‐529.

## CONSENT

Written informed consent was obtained from the patients to publish this report in accordance with the journal's patient consent policy.

## Data Availability

The data that support the findings of this study are available on request from the corresponding author. The data are not publicly available due to privacy or ethical restrictions.
